# Development of the PREDICT-Kidney online tool to promote informed decision-making about kidney cancer follow-up care: a qualitative co-design study

**DOI:** 10.1136/bmjopen-2025-110668

**Published:** 2026-04-16

**Authors:** Chiara Re, Georgia Stimpson, Grant D Stewart, Jack Bromley, Stephanie Archer, Carley Batley, Angela Godoy, Juliet Usher-Smith, Hannah Harrison

**Affiliations:** 1 Department of Surgery, University of Cambridge, Cambridge, UK; 2 IRCCS San Raffaele Hospital, Unit of Urology, Vita-Salute San Raffaele University, Milan, Italy; 3 Department of Public Health and Primary Care, University of Cambridge, Cambridge, UK; 4 CRUK Cambridge Centre, Cambridge Biomedical Campus, Cambridge, UK; 5 Department of Psychology, University of Cambridge, Cambridge, UK; 6 Department of Oncology, University of Cambridge, Cambridge, UK

**Keywords:** Patient-Centered Care, Kidney tumours, Research Design, Follow-Up Studies

## Abstract

**Abstract:**

**Objective:**

Co-design of the PREDICT-Kidney online tool by patients, members of the public and healthcare professionals (HCPs), to support the communication of the risk of recurrence following surgical treatment for kidney cancer.

**Design:**

Qualitative co-design study. Using an iterative process, feedback was collected (via workshops), prioritised and implemented.

**Setting:**

Online workshops with participants from across the UK were conducted between December 2023 and November 2024.

**Participants:**

18 adult participants, including patients surgically treated for kidney cancer, members of the public without a history of kidney cancer and HCPs involved in kidney cancer care.

**Primary and secondary outcomes:**

To produce an online tool to support the communication of risk of kidney cancer recurrence that is easy to use, easy to understand and acceptable to stakeholders. Secondary outcomes are the properties of the feedback collected, including volume and type.

**Results:**

Across nine workshops, 99 discrete feedback items were collected, resulting in 71 actionable changes to the initial prototype tool. Differences in priorities were observed between participant groups, especially around the inclusion of information about competing risks of death. Participants valued the tool for improving consistency of follow-up information, supporting shared decision-making and providing multiple visual formats to communicate risk. Iterative feedback led to refinements in terminology, design, content and delivery, including adjustments to the presentation of recurrence and mortality risk.

**Conclusions:**

A co-design approach was used to improve the PREDICT-Kidney online tool to align with the needs of patients and HCPs. A feasibility study is required to evaluate its use and impact in clinical practice.

**STRENGTHS AND LIMITATIONS OF THIS STUDY**

- Iterative co-design with patients, members of the public and healthcare professionals enhanced the relevance, usability and acceptability of the PREDICT-Kidney online tool.
- Recruitment across the UK via patient organisations, social media and professional networks captured diverse perspectives, but selection bias may have favoured participants with higher health literacy and healthcare professionals interested in risk communication.
- Workshops were recorded, transcribed and analysed independently by multiple researchers, ensuring rigour and transparency.
- Small sample size and recruitment from a single country may limit generalisability.
- The study did not assess real-world implementation or impact on patient decision-making.

##  Introduction

Approximately 20%–30% of patients surgically treated for a localised renal cell carcinoma (RCC) develop recurrence within 5 years.^1^ For this reason, surveillance, including interval imaging, is an important component of postoperative care.^2^ Several international guidelines^3^,^6^ recommend tailoring surveillance based on individual risk of recurrence, with follow-up schedules that vary in modality, frequency and duration depending on risk classification.^7^ Despite this, the details of the recommendations are imprecise, and the follow-up care delivery is not consistent across the UK.^8^ Moreover, surveillance is a source of anxiety for many patients and understanding of decision-making about follow-up remains poor.^9 10^ Our previous research involving patients with experience of kidney cancer follow-up identified improving and personalising surveillance as priorities for patients.^9^ Furthermore, recent studies showed that communication issues frequently lead to a lack of understanding regarding diagnosis, treatment and increase the psychosocial impact of kidney cancer.^11 12^ Online tools are becoming more widely used to support discussions between patients and clinicians, and to guide treatment recommendations.^13 14^ For example, the PREDICT Prostate^15^,^17^ and PREDICT Breast^18 19^ online tools have been shown to reduce decisional conflict when used in a clinical decision-making context and shift patient perception of prognosis to a more realistic level.^20^

In this study, we aimed to co-design the PREDICT-Kidney online tool: a new online tool for calculating and displaying the risk of recurrence of RCC alongside patients treated for kidney cancer, members of the public and healthcare professionals (HCPs) involved in kidney cancer care.

## Methods

To co-design the tool, we conducted a series of online workshops with three groups of participants: (1) patients surgically treated for kidney cancer; (2) members of the public without a history of kidney cancer and (3) HCPs involved in the follow-up care of patients with kidney cancer. The workshops were semistructured, allowing discussion among participants.

Patients were recruited via the charity Kidney Cancer UK, members of the public were recruited via social media, HCPs were recruited through professional networks within the research team and via the British Association of Urological Surgeons Oncology group. Following recruitment, which ran from December 2023 and January 2024, public participants completed a demographic questionnaire (age, gender, ethnicity, UK region). Patients provided demographics as well as the time since their first kidney cancer diagnosis. HCPs reported their current clinical role, years of experience and the region of the UK in which they work. Eligible participants received the study information sheet, a consent form and workshop details.

Before the first workshop, a prototype tool was designed and developed based on a tool built by the Winton Centre for Risk & Evidence Communication at the University of Cambridge for NHS Blood & Transplant and includes:

1. An introductory page, which provides a short introduction to the tool and its functionality.
2. The main page, where patient-specific characteristics can be entered, and a personalised risk of kidney cancer recurrence is displayed using a range of different visualisations.
3. A printed report, which summarises the personalised information about the risk of kidney cancer recurrence and provides more information about the patient-specific inputs.
4. Additional pages providing clinical and technical information about the tool (‘About’, ‘Legal’, ‘Publications’, ‘Technical’).

The tool calculates the risk of recurrence in the ten years following surgery using the Leibovich model,^21^ which is widely used to assess prognosis based on tumour characteristics following kidney cancer surgery in current clinical practice,^3^,^5^ adapted to include competing risk.^22^ This model is not appropriate for use with patients who have non-clear cell histological subtypes of kidney cancer, a known hereditary kidney cancer syndrome or metastatic disease.

We ran eight workshops (three with patients, three with members of the public and two with HCPs) between February 2024 and November 2024. All the workshops were led by a team consisting of two public health researchers (HH/GS), two urologists (CR/GDS) and an academic general practitioner (JU-S). In place of a third workshop with HCPs, feedback was sought via email.

In the first workshop with each group, members of the research team introduced the topic, presented the prototype version of the PREDICT-Kidney online tool (members of the public and patients were shown videos of clinicians using the tool to explain risk of recurrence to a patient, while HCPs were given the opportunity to test the tool themselves), and led a discussion about the design of the tool which covered usability, transparency and barriers to use.

The workshops were recorded using the Zoom conferencing software and subsequently transcribed and anonymised. Three researchers (CR/GS/HH) independently analysed the transcripts to identify and categorise perceived limitations and suggested changes to the prototype tool. One researcher (HH) then compiled the results into a list of potential actions, which were then discussed and prioritised by the wider research team based on request volume, relevance and technical feasibility. These actions were translated into implementation requirements for a web developer. Progress was tracked using a project management system (JIRA), and all changes were reviewed using a code review programme (GitHub). Once all requirements for that round of changes had been approved, an updated version of the PREDICT-Kidney tool was deployed.

In subsequent rounds of workshops, participants reviewed updated versions of the PREDICT-Kidney tool, and feedback was incorporated iteratively. The first round focused on general features of the tool; the second introduced information on risk of death from other causes and emphasised printed report features. The third explored use within urology clinics and guidance for clinicians using the tool for the first time.

Manuscript drafting and editing were conducted in accordance with the Standards for Reporting Qualitative Research reporting guideline^23^ and checklist^24^ (online supplemental material).

## Results

18 adults participated across eight workshops, each lasting approximately 90 min (table 1). Groups were balanced by gender and participants predominantly reported white ethnicity, with most HCPs being consultant urologists specialising in kidney cancer care with over 10 years’ experience. All three groups were generally positive about the tool, with several HCPs indicating that they would like to use it in their clinics. Participants in the patient group expressed that the tool would be helpful to standardise the information given to them by clinicians following surgery*—*“if there’s a tool like this available and its use is encouraged, it means that the delivery of the results to people will be more consistent” (Patient, round 1).

**Table 1:** Participant demographic characteristics

	HCPs	Patients	Public
No.	7	6	5
Gender			
Man	6 (85.7)	2 (33.3)	1 (20)
Woman	1 (14.3)	4 (66.7)	4 (80)
Race			
White		100 (100)	3 (60)
Black	–	0 (0)	1 (20)
Asian		0 (0)	1 (20)
Age			
<40		0 (0)	2 (40)
40–49		1 (16.7)	1 (20)
50–59	–	3 (50)	0 (0)
60–69		1 (16.7)	0 (0)
70–79		1 (16.7)	2 (40)
First kidney cancer diagnosis			
< 1 year ago		2 (33.3)	
1–2 years ago	–	3 (50)	–
>5 years ago		1 (16.7)	
Highest education level,			
Secondary education	–	–	1 (20)
University education			4 (80)
Current clinical role			
Urologists	3 (42.8)		
Oncologists	2 (28.6)	–	–
CNSs	2 (28.6)		
Year of experience in that role			
<1	0 (0)		
1–5	1 (14.3)		
5–10	1 (14.3)	–	–
>10	5 (71.4)		
Living/working place			
England, East	3 (42.8)	2 (33.3)	1 (20)
England, West Midlands	0 (0)	1 (16.7)	1 (20)
England, South East	0 (0)	2 (33.3)	2 (40)
England, South West	2 (28.6)	0 (0)	0 (0)
England, North East	0 (0)	1 (16.7)	0 (0)
England, London	2 (28.6)	0 (0)	1 (20)

Categorical data are presented as n (%).

CNSs, clinical nurse specialists; HCPs, healthcare professionals.

Across all workshops, 99 discrete feedback items were identified: patients (n=42), HCPs (n=41), members of the public (n=37) and the research team (n=18). Feedback was grouped by topic: terminology/clarity (n=25), design (n=23), acceptability (n=20), new features (n=18), errors (n=12) and site infrastructure (n=1). 92 items led to proposed changes, with 71 (72%) implemented after prioritisation based on relevance, feasibility and difficulty of implementation. Full details of each feedback item, including motivating quotes, proposed actions, prioritisation, justification if not implemented and changes made, are listed in online supplemental table 1 and summarised in table 2.

**Table 2:** Summary of feedback

Part of website	Items of feedback																	
	Total	Round			Request made by?*				Theme						Priority†			Resulting actions
		1	2	3	Patients	Public	HCP	Research team	Acceptability	Additions	Display/design	Errors	Infrastructure	Terminology/clarity	High	Medium	Low	
Infrastructure	1	1	0	0	0	0	0	1	0	0	0	0	1	0	1	0	0	1
Introductory page	22	5	8	9	5	7	10	9	1	1	3	6	0	12	5	7	10	21
Inputs	20	14	3	3	7	8	9	2	0	5	3	1	0	10	4	7	9	13
Results	24	10	6	8	14	9	10	1	5	6	8	2	0	1	2	8	10	16
Competing risks	12	2	7	3	6	5	7	2	5	4	3	0	0	0	2	7	3	10
Printed report	16	2	9	5	8	8	3	4	2	2	7	2	0	3	5	4	7	15
User guide	2	0	0	2	0	0	2	0	2	0	0	0	0	0	0	0	1	1
*General and other*	5	3	0	2	3	2	2	1	3	0	1	1	0	0	2	1	0	3

* Numbers in this section may not sum to the total, as requests were sometimes made by multiple groups.

† Numbers in this section may not sum to total, as feedback not requiring action was not prioritised

HCPs, healthcare professionals.

**Figure 1:**
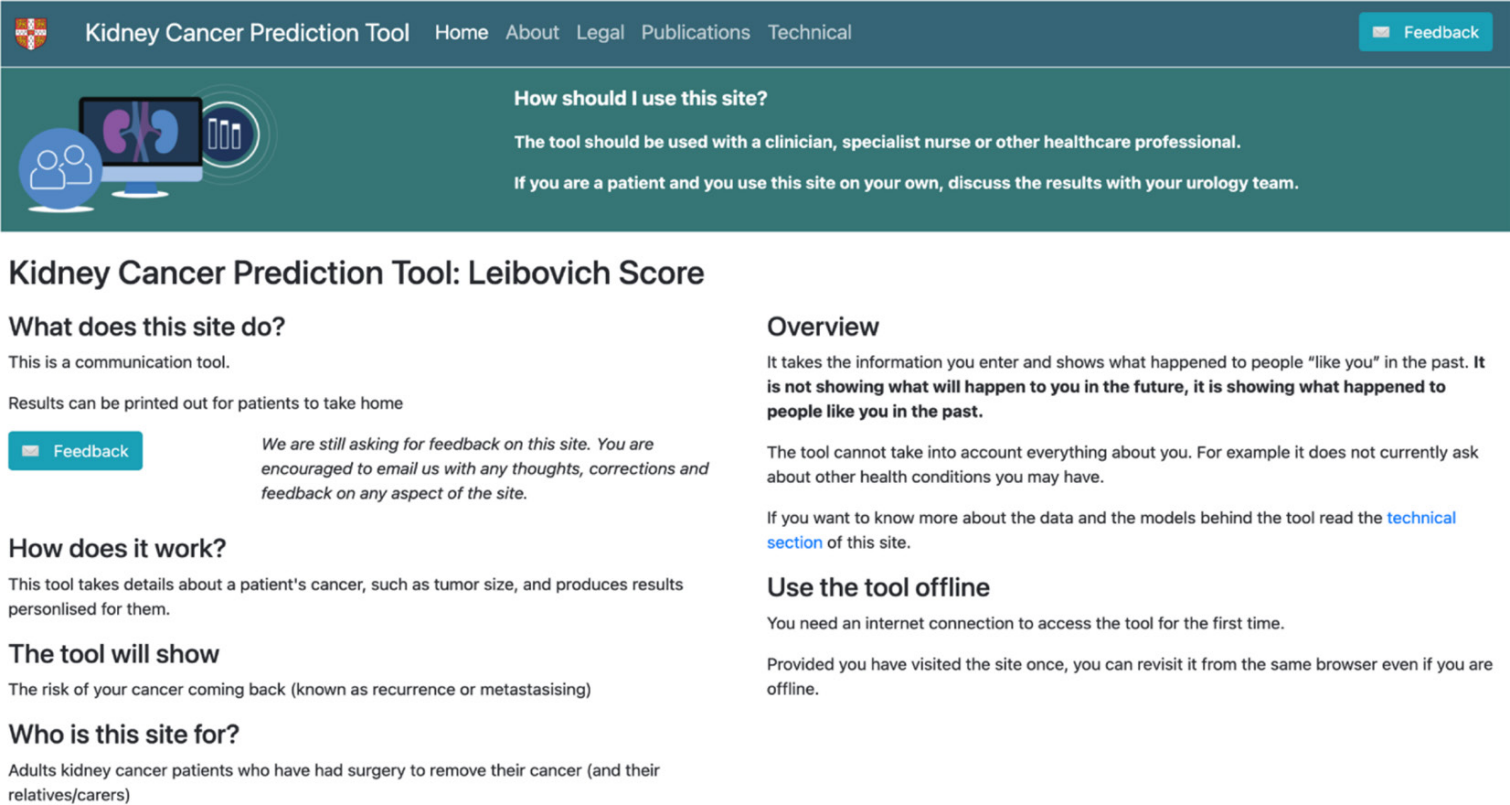
First version of the introductory page.

**Figure 2:**
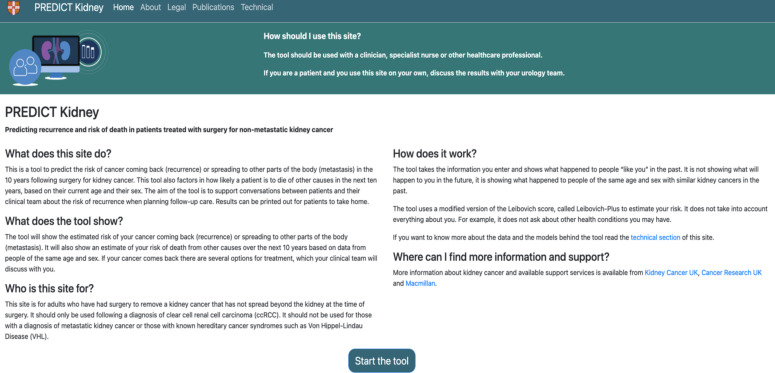
Final version of the introductory page.

Feedback addressed different sections of the tool. For the introductory page (n=22) (figure 1), participants requested clearer definitions of terms such as recurrence, “…you said recurrence means coming back. I don’t know the meaning of recurrence…” (Patient, round 1) and metastasis *“*you write the term ‘metastasis’ for this one*.* It means the cancer is spreading to other parts of the body?” (Member of the public, round 2). Additionally, HCPs suggested including patient resources and information on treatment options “…[you should] be able to link directly from the tool to patient information, patient charities.” (HCP, round 1) (figure 2).

**Figure 3:**
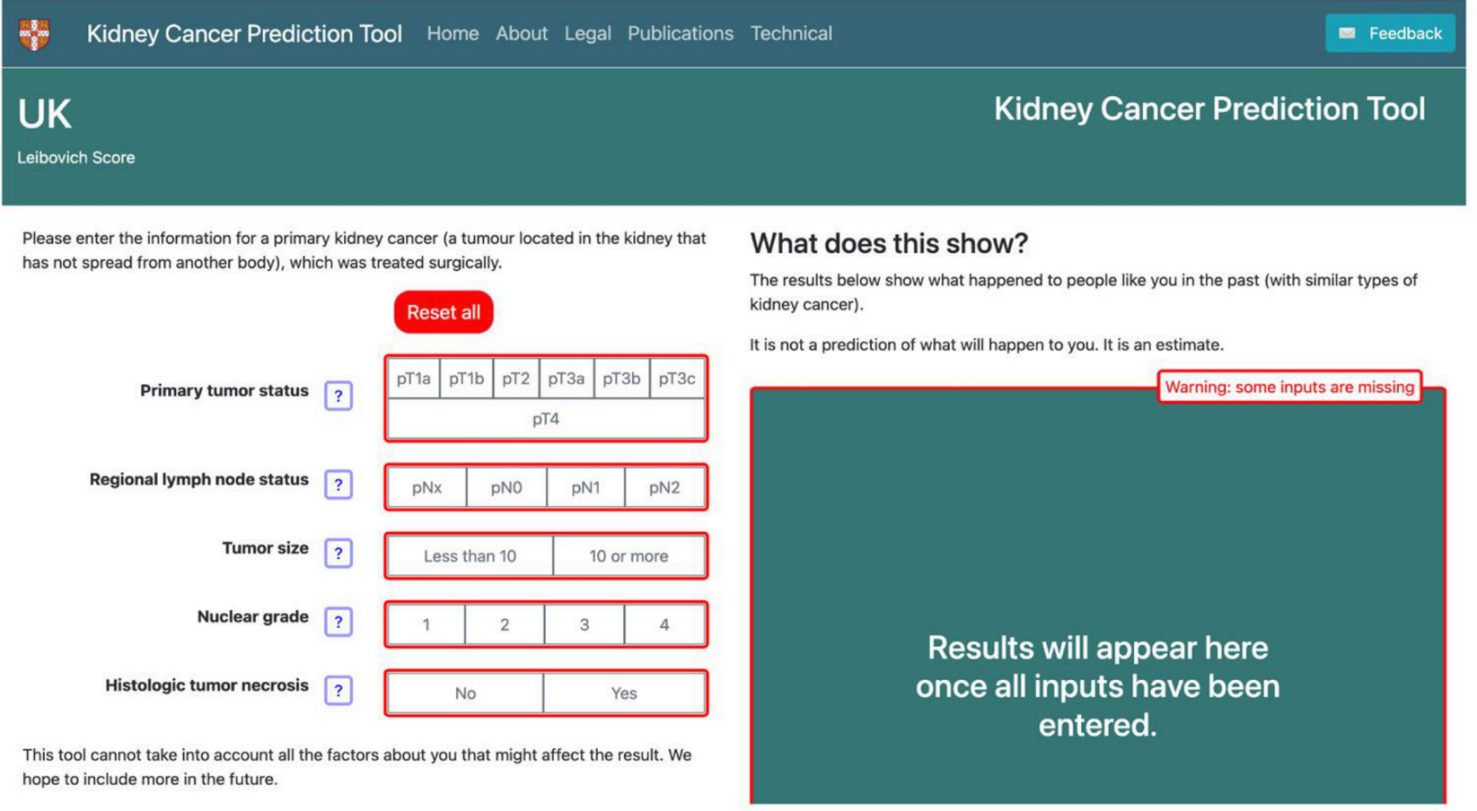
First version of the patient characteristics input section.

**Figure 4:**
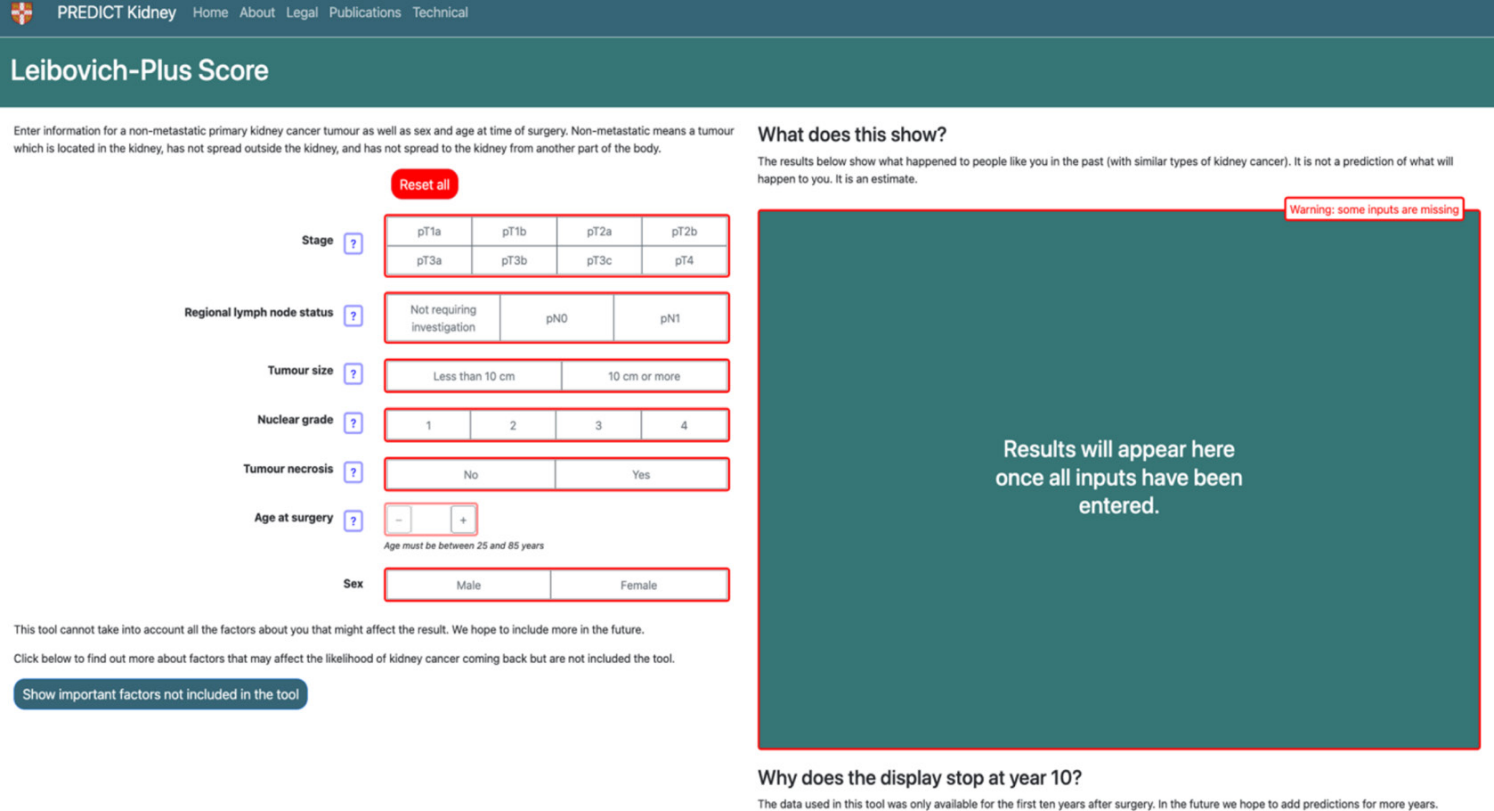
Final version of the patient characteristics input section.

For the input section (n=20) (figure 3), the most frequent requests were about terminology or clarity (n*=*10), or suggesting additional features (e.g. genetics or familial risk factors, patient characteristics) (n*=*5). The description of necrosis and lymph node status was poorly understood by both patients and the public, and considered hard to explain by HCPs, especially when lymph node status was unknown. “Necrosis doesn’t mean anything to me. And also, the regional lymph node status” (Member of the public, round 1)*. “*Dead cancer cells sounds like a good thing rather than a bad thing and trying to explain why their risk is higher with necrosis*…*” (HCP, round 2). The final phrasing agreed on across all three groups for necrosis was “The tumour necrosis indicates if dead cancer cells were found in the samples removed at surgery. Dead cells may indicate a faster-growing tumour. If necrosis was detected the cancer is more likely to return”. The description for lymph node status when it was unknown, was changed to “No investigation required (pNx)—There were no lymph nodes in the tissue removed at surgery. This is common if there are no noticeable lymph nodes present” (figure 4).

**Figure 5:**
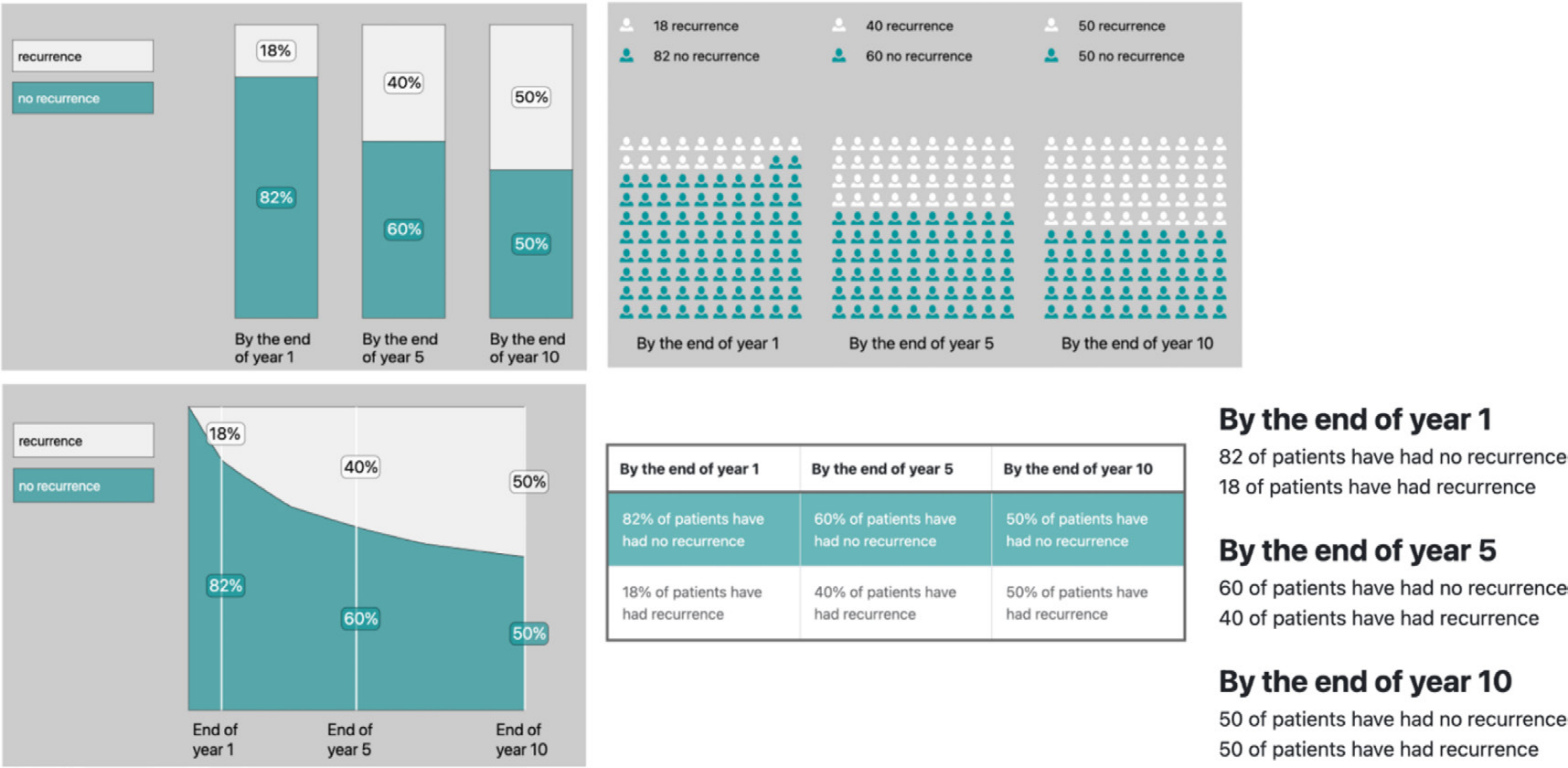
First version of the interpretation of the results section.

**Figure 6:**
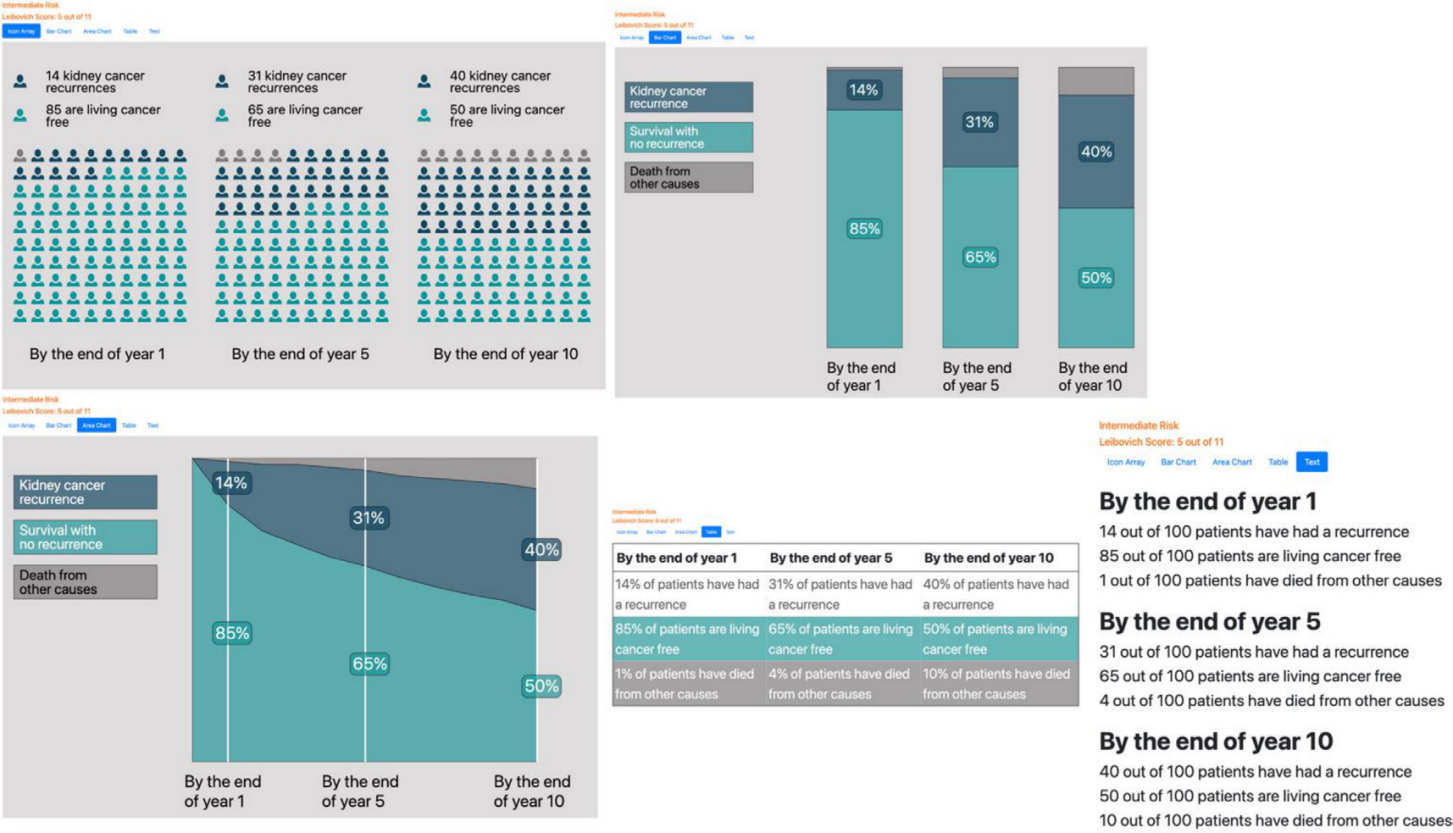
Final version of the interpretation of the results section.

In the results section (n=24) (figure 5), all three groups appreciated the different visual formats that provide the same information (icon array, bar chart, area chart, table and text), with the consensus being that the icon array was the easiest visual to interpret *“*The image with the people [icon array] felt less scary than the image with the bar graph.” (Member of the public, round 1) (figure 6). Moreover, patients expressed concern about the results potentially being likely to ‘scare’ or ‘alarm’. In response to this, the size text of the heading describing the risk group (eg, Intermediate Risk, Leibovich Score 5 out of 11) was decreased, and a button was added to ensure the results were not revealed until the clinician had prepared their patient for the risk information (figure 6). We also included in the HCP user guide the recommendation to assess the readiness of each patient to receive information about recurrence risk and focus initially on the number of people living cancer-free 10 years postsurgery.

12 items of feedback were related to how best to include death from other causes in the results (n=7). HCPs were generally enthusiastic, noting it added valuable context and personalisation to recurrence risk. “I think it is a very powerful tool to make sure the patient’s general health is being considered” (HCP, round 2). Patients and members of the public agreed it could provide useful context when making decisions about appropriate follow-up care (round 2), but when this was implemented in the tool and presented in a mock consultation (round 3), several participants questioned its value “I don’t see the point of putting in people who’ve died of something else” (Patient, round 3), and others expressed discomfort with the increased focus on risk of death from other causes “It is very different - as you say, if they’ve come out of the operation and this is the first time it’s [the cancer diagnosis] confirmed, that’s quite a lot to take in in itself, isn’t it? …” (Member of the public, round 3).

Following this feedback, we made substantial changes to balance the needs of the different stakeholders. We retained the competing risks calculation but reduced emphasis on death by simplifying labels, adjusting graphic colours (figure 6), and adding guidance for HCPs to focus on recurrence rather than mortality, particularly at the first follow-up appointment.

16 items of feedback were specific to the printed report designed to be provided to patients following their consultation. Feedback focused on ensuring legibility in black-and-white printing and improving access to resources when hyperlinks were not clickable “…anything you get printed out from the NHS will not be in colour.” (Patient, round 2) and making it easier to access resources when links can’t be clicked on “…of course if you’ve got a handprinted, you wouldn’t be able to click on it [the url] to do it but at least you’ve got an idea of what keywords to search for.” (Patient, round 2) (online supplemental figure 1).

## Discussion

This paper details the co-design of an online tool for communicating recurrence risk during follow-up care for patients who have undergone surgery for kidney cancer. Working together in an interactive process with members of the public, patients who had been treated for kidney cancer and HCPs involved in the care of those with kidney cancer, we identified user requirements for the tool and have developed a tool that all felt would be useful within clinical care. The value of the co-design approach is illustrated by the significant modifications made across all areas of the prototype tool in response to feedback and reinforces the need for co-design when developing new disease-specific tools. Notably, our findings have broader relevance for improving recurrence risk communication in other cancer types as well. For example, uncertainties regarding the interpretation of necrosis and lymph node status emerged as particularly relevant in kidney cancer but are common across many solid tumours. The inclusion of HCPs, patients and members of the public further revealed important differences in priorities and expectations and underlined the importance of including diverse stakeholders in the design process. What clinicians may see as sufficient or appropriate information can differ substantially from what patients perceive as meaningful or supportive, mainly based on their personal experience. Similar conclusions were drawn by Adam *et al*, who used a co-design approach to develop the Structured Personalised Assessment for Reviews After Cancer tool.^14^ In that study, patients, clinicians, digital and computing science experts were involved in two workshops, highlighting the value of gathering extensive input from stakeholders with diverse disciplinary backgrounds and perspectives to collect a large amount of meaningful data.^14^

Our experience also illustrates the importance of an iterative co-design process. Multiple rounds of feedback allowed time for reflection and refinements, further clarification, helping to reveal concerns that did not surface initially and ensuring that issues were identified and addressed progressively. A clear example is the inclusion of competing risk of death information: while patients accepted this concept when discussed in abstract terms (round 2), their response shifted when the same information was presented in a simulated clinical interaction. The presentation in this context made several participants uncomfortable, highlighting a gap between intellectual understanding and emotional impact (round 3). This insight led to substantial changes in how the competing risks information was framed and communicated, specifically by de-emphasising it in the visual presentation. The need for structured, iterative co-design is further supported by Donoso *et al*, who used an eight-step co-design process to adapt the CanRisk tool for communicating multifactorial breast and ovarian cancer risk.^25 26^ Although CanRisk is now increasingly used in routine care, its original format was not suitable for patients, members of the public and several HCPs in diverse settings. The co-design approach broadened the diversity of ideas and ensured the report format was clear, acceptable and minimised the risk of misinterpretation.^19^ Furthermore, repeated rounds of feedback improved usability and reduced the likelihood of errors.^27^

Another strength of our approach was the use of a prioritisation framework to translate feedback into targeted, feasible changes. A key challenge in any co-design process is resolving conflicting suggestions or feedback that falls outside the project scope. By systematically evaluating each recommendation for relevance, feasibility and frequency, we were able to make transparent, balanced decisions about which modifications to implement. Despite these strengths, our study has some limitations. The relatively small sample size and recruitment from a single country (UK) may limit generalisability. While we successfully recruited patients with a range of experiences and diagnoses, members of the public with limited knowledge of kidney cancer and HCPs, our recruitment strategy may have introduced selection bias, particularly favouring patients and members of the public with a high level of health literacy and HCPs with an interest and experience in delivering risk information.

A feasibility study is being conducted in three hospital centres across the UK, which will test the acceptability and usability of the tool within its intended clinical pathway. The feasibility study will recruit a larger group of patients and clinicians and aims to include a more diverse group of patients.^28^ In the longer term, we hope to also expand the tool to include information on the individualised benefits and harms of surveillance and adjuvant treatment to support shared decision-making and reduce unnecessary investigations.

## Conclusions

The PREDICT-Kidney web tool is a promising tool to improve the current follow-up care pathway for patients treated for kidney cancer. Its core innovations include the integration of personalised recurrence risk with competing risk of death, multiple visual formats to support patient understanding, and a printed report for shared decision-making. The iterative co-design process with patients, members of the public and HCPs enhanced the tool’s relevance, usability and potential acceptability, providing a strong foundation for future testing in real-world clinical settings.

The co-design process played a central role in shaping and improving its development, strengthening the tool’s relevance, usability and potential acceptability, laying a strong foundation for future testing in clinical settings.

## Supplementary material

10.1136/bmjopen-2025-110668 online supplemental file 1

10.1136/bmjopen-2025-110668 online supplemental file 2

10.1136/bmjopen-2025-110668 online supplemental file 3

## Data Availability

All data relevant to the study are included in the article or uploaded as supplementary information.
