# Supplementary material for: Development of the PREDICT-Kidney online tool to promote informed decision-making about kidney cancer follow-up care: a qualitative co-design study
Source: BMJ Open. 2026 Apr 16;16(4):e110668. doi: 10.1136/bmjopen-2025-110668 (PMC13110639; doi:10.1136/bmjopen-2025-110668)
Supplement: online supplemental file 1 [file bmjopen-16-4-s001.pdf]

Patient details:
Name:
NHS number:
Date of birth:

Consultant:
Name:
Date: 1 July 2025
Signature:

PREDICT Kidney is a prognostic tool to predict recurrence in patients surgically treated for non-metastatic kidney cancer

Results: Intermediate Risk, Leibovich Score 5 out of 11

**Stage**	pT2b
**Regional lymph node status**	pN1
**Tumour size**	Less than 10 cm
**Nuclear grade**	2
**Tumour necrosis**	No
**Age at surgery**	65
**Sex**	Female

Based on the details of your tumour, you are at **intermediate risk** of your cancer coming back or spreading. The estimated risk of the cancer coming back (recurrence) or spreading to other parts of the body (metastasis) in the 5 years following surgery is **31%** based on what has happened previously to people of the same age and sex and with the same type of tumour.

In other words, **the cancer will come back or spread in the 5 years following surgery in about 31 out of 100 patients of the same age and sex with a similar tumour.**

The following images show that risk in different ways. They also show the estimated risk of the cancer coming back or spreading between 1 year and 10 years.

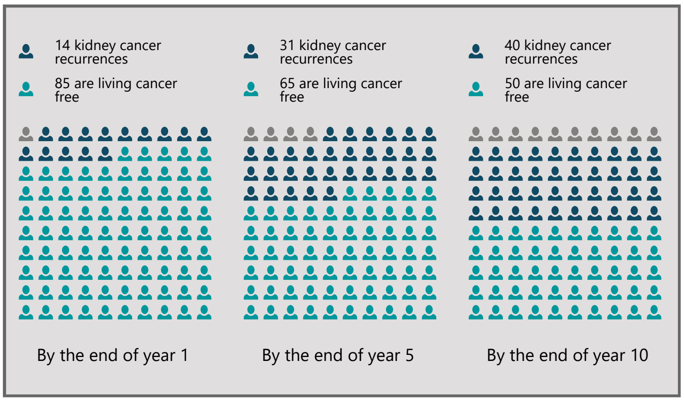

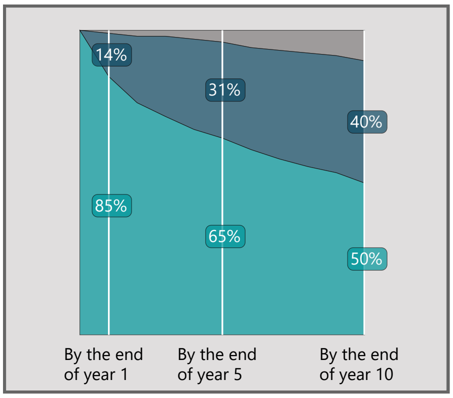

By the end of year 1	By the end of year 5	By the end of year 10
14% of patients have had a recurrence	31% of patients have had a recurrence	40% of patients have had a recurrence
85% of patients are living cancer free	65% of patients are living cancer free	50% of patients are living cancer free

Kidney cancer recurrence

Survival with no recurrence

**Patient details:**	**Consultant:**
Name:	Name:
NHS number:	Date: 1 July 2025
Date of birth:	Signature:

Further details

Stage - The pathological stage of a kidney cancer tumour is a measure of its size and how far it has spread. This is determined by assessing cancer tissue removed during surgery.

Your tumour stage is **pT2b** - the cancer is larger than 10cm and only inside the kidney

Regional lymph node status - The regional lymph node status indicates if the cancer has spread to lymph nodes near the kidney. Lymph nodes are a network of glands found throughout the body that drain away waste products and fight infections. Lymph nodes near the kidney may be removed during surgery and tested for the presence of cancer. However, it is common for no lymph nodes to be removed at the time of surgery and no further investigation to be required.

Your regional lymph node status is **pN1** - cancer cells were detected in one or more lymph nodes near the tumour

Tumour size - The size of the kidney cancer tumour removed during surgery.

Your tumour is **less than 10 cm** in size

Nuclear grade - The nuclear grade is a scale indicating how much the cancer cells look like normal cells. This is sometimes called the Fuhrman scale. Kidney cancers are graded 1 to 4. Grade 1 is the lowest (the most like normal cells) and grade 4 is the highest (the least like normal cells). Higher grade cancers tend to grow more quickly and are more likely to spread to other parts of the body.

Your nuclear grade is **2**

Tumour necrosis - The tumour necrosis indicates if dead cancer cells were found in the samples removed at surgery. Dead cells may indicate a faster-growing tumour. If necrosis was detected the cancer is more likely to return.

Your tumour had **no necrosis**

MORE INFORMATION AND SUPPORT:

- Kidney Cancer UK - https://www.kcuk.org.uk/
- Cancer Research UK - https://www.cancerresearchuk.org/about-cancer/kidney-cancer
- Macmillan - https://www.macmillan.org.uk/cancer-information-and-support/kidney-cancer
