# Supplementary material for: Development of the PREDICT-Kidney online tool to promote informed decision-making about kidney cancer follow-up care: a qualitative co-design study
Source: BMJ Open. 2026 Apr 16;16(4):e110668. doi: 10.1136/bmjopen-2025-110668 (PMC13110639; doi:10.1136/bmjopen-2025-110668)
Supplement: online supplemental file 3 [file bmjopen-16-4-s003.docx]

The SRQR reporting checklist

For checking that qualitative health research articles can be understood and used by everyone

|  | Item Description | Location (or reason for not reporting) |
| --- | --- | --- |
| **Title & Abstract** |  |  |
| [Title](https:/resources.equator-network.org/reporting-guidelines/srqr/items/title.html) | Co-design of the PREDICT-Kidney online tool to promote informed decision-making about kidney cancer follow-up care | Page 2 |
| [Abstract](https:/resources.equator-network.org/reporting-guidelines/srqr/items/abstract.html) | **Objectives:** To co-design the PREDICT-Kidney online tool with patients, members of the public and healthcare professionals (HCPs) to support communication about risk of kidney cancer recurrence in patients treated surgically for the disease.  **Methods:** We conducted three sets of workshops in which patients, members of the public and HCPs provided iterative rounds of feedback regarding the PREDICT-Kidney online tool. The tool was shown to, and in some cases tested by, participants. Feedback was solicited on overall design, clarity and potential barriers to use. Transcripts of each workshop were analysed to identify areas of potential improvement, which were then prioritised based on both relevance and technical difficulty and presented for further feedback in later workshops.  **Results:** 18 participants took part across the three sets of workshops. We received 99 discrete items of feedback, which resulted in 71 actionable changes made to the initial prototype. Differences in priorities were observed between participant groups, especially between patients and HCPs. For example, patients had varying reactions to the inclusion of individualised competing risk of death, whereas HCPs consistently supported including other-cause mortality. All participants were positive about the potential of the new online tool to improve follow-up care.  **Discussion:** This co-design study shows the value of collaboration between HCPs and patients when designing communication of complex health information and highlights areas of communication that need particular consideration.  **Conclusions:** Co-designing the PREDICT-Kidney online tool improved its usability and alignment with user needs. A feasibility study is needed to evaluate its use in clinical practice. | Page 3 |
| **Introduction** |  |  |
| [Problem Formulation](https:/resources.equator-network.org/reporting-guidelines/srqr/items/problem-formulation.html) | Follow-up for surgically treated kidney cancer can be challenging, partly due to the difficulty of clearly communicating complex prognostic information between healthcare providers and patients. This may contribute to patient anxiety and uncertainty about their care. Online tools are increasingly recognised as valuable in supporting clinician perceptions and treatment recommendations, potentially reducing decisional conflict when used in clinical decision-making (e.g. PREDICT Prostate and PREDICT Breast tools). While risk models are widely recommended in follow-up guidelines, there is still no standardised approach for conveying prognostic information - particularly regarding the competing risks of death from other causes - leaving an important gap in available resources and in the rationale underlying follow-up strategies. | Page 5 |
| [Purpose or research question](https:/resources.equator-network.org/reporting-guidelines/srqr/items/purpose.html) | The purpose of our study is to co-design the PREDICT-Kidney online tool with patients, members of the public and healthcare professionals to support communication about risk of kidney cancer recurrence and competing risks of death following surgery for kidney cancer. | Page 5 |
| **Methods** |  |  |
| [Qualitative approach and research paradigm](https:/resources.equator-network.org/reporting-guidelines/srqr/items/qualitative-approach.html) | We used a co-design methodology with iterative workshops involving patients, members of the public and healthcare professionals. This design approach is particularly well-suited to the development of digital health tools, as it enables stakeholders to contribute actively to shaping functionality, design, and content. Data comprised transcripts of workshop discussions, supplemented by written feedback and observations from the research team. A thematic content analysis was applied to identify user needs, concerns, and priorities, which were then translated into technical and design requirements. | Page 6 |
| [Researcher characteristics and reflexivity](https:/resources.equator-network.org/reporting-guidelines/srqr/items/researcher-characteristics-and-reflexivity.html) | Our multidisciplinary team included urologists with extensive experience in the management of kidney cancer, a general practitioner, two researchers with expertise in psychology, behavioural science, and risk communication and a web developer. This range of clinical and methodological expertise allowed us to bring different perspectives to the co-design process. | Page 7 |
| [Context](https:/resources.equator-network.org/reporting-guidelines/srqr/items/context.html) | The study was conducted in the UK via online workshops (Zoom) to maximise accessibility for patients, healthcare professionals, and public participants recruited through Kidney Cancer UK, professional networks, and social media. The UK setting is relevant given the variability of kidney cancer follow-up pathways and the absence of standardised communication practices, which may have influenced participant perspectives. | Page 7 |
| [Sampling strategy](https:/resources.equator-network.org/reporting-guidelines/srqr/items/sampling-strategy.html) | Patients were recruited via the charity Kidney Cancer UK (KCUK), members of the public were recruited via social media, HCPs were recruited through professional networks within the research team and via the British Association of Urological Surgeons Oncology group. Following recruitment, which ranged between December 2023 and January 2024, public participants completed a demographic questionnaire, patients provided demographics as well as the time since their first kidney cancer diagnosis. HCPs reported their current clinical role, years of experience and the region of the UK in which they work. | Page 6 |
| [Ethical issues pertaining to human subjects](https:/resources.equator-network.org/reporting-guidelines/srqr/items/ethics.html) | Ethical approval was obtained from the Department of Psychology Research Ethics Committee at the University of Cambridge (Ref. 8171.214). | Page 6 |
| [Data collection methods](https:/resources.equator-network.org/reporting-guidelines/srqr/items/data-collection-methods.html) | Qualitative data were collected from three iterative workshops involving patients, healthcare professionals, and members of public between February 2024 and November 2024. Workshops combined discussion, observation, and document review, with procedures adapted based on emerging insights to clarify and refine content. Triangulating multiple participant groups and methods ensured diverse perspectives informed iterative tool development. | Page 7-8 |
| [Data collection instruments and technologies](https:/resources.equator-network.org/reporting-guidelines/srqr/items/data-collection-instruments.html) | The workshops were recorded using the Zoom audio/video conferencing software. | Page 7 |
| [Units of study](https:/resources.equator-network.org/reporting-guidelines/srqr/items/units-of-study.html) | We ran eight workshops (three with patients, three with members of the public and two with healthcare professionals). In place of a third workshop with healthcare professionals, feedback was sought via email. Eighteen adults took part across the eight workshops, with each workshop lasting an average of 90 minutes.  The groups resulted in a relatively equal distribution of men and women; most reported their ethnicity as “White”. Across healthcare professionals, most were consultant urologists specialising in kidney cancer care with more than ten years of experience in that role (participants demographic characteristics are listed in **Table 1**). | Page  Page 20 (Table 1) |
| [Data processing](https:/resources.equator-network.org/reporting-guidelines/srqr/items/data-processing.html) | The workshops were recorded using the Zoom conferencing software and subsequently transcribed and anonymised. Three researchers independently analysed the transcripts to identify and categorise perceived limitations and suggested changes to the prototype tool. One researcher then compiled the results into a list of potential actions, which were then discussed and prioritised by the wider research team based on request volume, relevance and technical feasibility. These actions were translated into implementation requirements for a web developer. Progress was tracked using a project management system (JIRA), and all changes were reviewed using a code review program (GitHub). Once all requirements for that round of changes had been approved, an updated version of the PREDICT-Kidney tool was deployed. | Page 8 |
| [Data analysis](https:/resources.equator-network.org/reporting-guidelines/srqr/items/data-analysis.html) | Three researchers independently coded transcripts, then collaboratively developed themes through iterative discussion. This approach was chosen to ensure rigorous, reflexive interpretation and to capture diverse perspectives. | Page 7 |
| [Techniques to enhance trustworthiness](https:/resources.equator-network.org/reporting-guidelines/srqr/items/trustworthiness.html) | Trustworthiness was strengthened through independent double-checking of transcripts by three research team members, ensuring that the findings accurately reflected participants’ perspectives and enhancing transparency and rigor. | Page 7-8 |
| **Results** |  |  |
| [Synthesis and interpretation](https:/resources.equator-network.org/reporting-guidelines/srqr/items/synthesis-and-interpretation.html) | 18 participants took part across the three sets of workshops. We received 99 discrete items of feedback, which resulted in 71 actionable changes made to the initial prototype. Differences in priorities were observed between participant groups, especially between patients and healthcare professionals. For example, patients had varying reactions to the inclusion of individualised competing risk of death, whereas healthcare professionals consistently supported including other-cause mortality. All participants were positive about the potential of the new online tool to improve follow-up care.  (Full details of each feedback item, including motivating quotes, proposed actions, prioritisation, justification if not implemented, and a change log of alterations to the online tool, are listed in **Supplementary Table 1** and summarised in **Table 2**). | Page 8-12  Page 21 (Table 2)  Supplementary materials (Supplementary Table 1) |
| [Links to empirical data](https:/resources.equator-network.org/reporting-guidelines/srqr/items/links-to-empirical-data.html) | Not applicable |  |
| **Discussion** |  |  |
| [Integration with prior work, implications, transferability, and contribution(s) to the field](https:/resources.equator-network.org/reporting-guidelines/srqr/items/integration-with-prior-work.html) | This paper details the co-design of the PREDICT-Kidney online tool for communicating recurrence risk during follow-up care for patients who have undergone surgery for kidney cancer. Working together in an interactive process with members of the public, patients who had been diagnosed with kidney cancer, and healthcare professionals involved in the care of those with kidney cancer, we identified user requirements for the tool and have developed a tool that all felt would be useful within clinical care.  The PREDICT-Kidney online tool has the potential to improve patients’ understanding of their recurrence risk and optimise benefits for patients (earlier detection of recurrent disease, reduction in imaging for low-risk individuals) and the healthcare system (more efficient resource use). Future versions of the tool could also be developed to inform decisions around adjuvant treatment. | Page 12-13 |
| [Limitations](https:/resources.equator-network.org/reporting-guidelines/srqr/items/limitations.html) | The relatively small sample size and recruitment from a single country (UK) may limit generalisability. While we successfully recruited patients with a range of experiences and diagnoses, members of the public with limited knowledge of kidney cancer and healthcare professionals, our recruitment strategy may have introduced selection bias, particularly favouring patients and members of the public with a high level of health literacy and healthcare professionals with an interest and experience in delivering risk information. | Page 14 |
| **Other** |  |  |
| [Conflicts of interest](https:/resources.equator-network.org/reporting-guidelines/srqr/items/conflicts-of-interest.html) | Grant D. Stewart has received educational grants from AstraZeneca; consultancy fees from Evinova and Qurin; travel expenses from MSD; he is Clinical lead (urology) National Kidney Cancer Audit and Topic Advisor for the NICE kidney cancer guideline.  All the other co-authors have no competing interests to declare. | Page 23 |
| [Funding](https:/resources.equator-network.org/reporting-guidelines/srqr/items/funding.html) | This project was funded by the National Institute for Health and Care Research (NIHR) under its Research for Patient Benefit (RfPB) Programme (Grant Reference Number NIHR205404).  HH is funded by the CRUK International Alliance for Cancer Early Detection (ACED) Pathway Award (EDDAPA-2022/100001).  GDS is supported by The Mark Foundation for Cancer Research [RG95043], the Cancer Research UK Cambridge Centre [C9685/A25177 and CTRQQR-2021\100012] and NIHR Cambridge Biomedical Research Centre (NIHR203312).  The views expressed are those of the author(s) and not necessarily those of the NIHR or the Department of Health and Social Care. | Page 23 |
