# Supplementary material for: Development of the PREDICT-Kidney online tool to promote informed decision-making about kidney cancer follow-up care: a qualitative co-design study
Source: BMJ Open. 2026 Apr 16;16(4):e110668. doi: 10.1136/bmjopen-2025-110668 (PMC13110639; doi:10.1136/bmjopen-2025-110668)
Supplement: Supplementary file 4 [file bmjopen-2025-110668.reviewer_comments.pdf]

PEER REVIEW HISTORY

BMJ Open publishes all reviews undertaken for accepted manuscripts. Reviewers are asked to complete a checklist review form and are provided with free text boxes to elaborate on their assessment. These free text comments are reproduced below.

ARTICLE DETAILS

Title (Provisional)

Development of the PREDICT-Kidney online tool to support informed decision-making about kidney cancer follow-up care: a qualitative co-design study

Authors

Re, Chiara; Stimpson, Georgia; Stewart, Grant D; Bromley, Jack; Archer, Stephanie; Batley, Carley; Godoy, Angela; Usher-Smith, Juliet; Harrison, Hannah

---

VERSION 1 - REVIEW

**Reviewer**	**1**
**Name**	**Weibing, Shuang**
**Affiliation**	**The first hosptial of shanxi medical university**
**Date**	**20-Nov-2025**
**COI**	**None**

This study describes how the research team co-designed the PREDICT-Kidney online tool through multiple rounds of workshops involving post-operative kidney cancer patients, the general public, and healthcare professionals to support communication about kidney cancer recurrence risk. During the process, 99 pieces of feedback were collected, based on which 71 feasible modifications were made to the tool prototype. The study also indicates that the tool has been recognized by participants, laying a foundation for subsequent clinical feasibility research and providing a reference for the design of other communication tools for cancer recurrence risk. The study is scientifically designed, aligned with user needs, and its outcomes hold both clinical and academic value.

However, the manuscript requires focused attention and improvements on the following issues:

1.Supplement mitigation strategies for limitations in the discussion: Sentences 321-324 mention the limitations of "small sample size and recruitment from a single country". It is recommended to add "how to address these issues in future research", for example, "Subsequent feasibility studies plan to expand to 3 countries, increase the sample size to over 100, and include populations with low health literacy", to enhance the logical continuity of the research.

2.The conclusions in Sentences 327-330 only state that "the tool lays a foundation for clinical testing". It is recommended to supplement "the core innovations of the tool and its differences from existing similar tools" to strengthen the academic value of the research.

**Reviewer**	**2**
**Name**	**Lawrentschuk , Nathan**
**Affiliation**	**University of Melbourne, Surgery**
**Date**	**26-Nov-2025**
**COI**	**None**

Overall good paper but please shorten.

Also the bigger question that should be part of this is limiting excessive investigations and follow-up- the minimum follow-up is generally the best. The EAU states for Grade1-2 T1a RCC 3 years, other guidlines say 5- we need clear direction as well as prognosis becuase this tool could easily mislead patients to wanting mnore follow-up please comment....

Surely AI has a role here rathger than etering of data?

Also needs to lose 400 words or so to make punchier.

VERSION 1 - AUTHOR RESPONSE

Reviewer: 1
Dr. Shuang Weibing, The first hosptial of shanxi medical university
Comments to the Author:
This study describes how the research team co-designed the PREDICT-Kidney online tool through multiple rounds of workshops involving post-operative kidney cancer patients, the general public, and healthcare professionals to support communication about kidney cancer recurrence risk. During the process, 99 pieces of feedback were collected, based on which 71 feasible modifications were made to the tool prototype. The study also indicates that the tool has been recognized by participants, laying a foundation for subsequent clinical feasibility research and providing a reference for the design of other communication tools for cancer recurrence risk. The study is scientifically designed, aligned with user needs, and its outcomes hold both clinical and academic value.

We thank the reviewer for their comments on the manuscript.

However, the manuscript requires focused attention and improvements on the following issues:

1. *Supplement mitigation strategies for limitations in the discussion: Sentences 321-324 mention the limitations of "small sample size and recruitment from a single country". It is recommended to add "how to address these issues in future research", for example, "Subsequent feasibility studies plan to expand to 3 countries, increase the*

sample size to over 100, and include populations with low health literacy", to enhance the logical continuity of the research.

We agree that information about mitigating the limitations would improve the manuscript. We are currently conducting a feasibility study in which the PREDICT-Kidney tool will be used in three UK hospital clinics. This will expose the tool to a larger number of patients and clinicians and aims to include a more diverse range of patients (e.g. with varying levels of health literacy) (1).

We have added the following text to the Discussion (line 361): "**A feasibility study is being conducted in three hospital centres across the UK, which will test the acceptability and usability of the tool within its intended clinical pathway (1). The feasibility study will recruit a larger group of patients and clinicians and aims to include a more diverse group of patients**."

2. *The conclusions in Sentences 327-330 only state that "the tool lays a foundation for clinical testing". It is recommended to supplement "the core innovations of the tool and its differences from existing similar tools" to strengthen the academic value of the research.*

We thank the Reviewer for this suggestion. We have revised the conclusion to highlight the core innovations of the PREDICT-Kidney tool and how it differs from existing tools (line 370).

"The PREDICT-Kidney web tool is a promising tool to improve the current follow-up care pathway for patients treated for kidney cancer. **Its core innovations include the integration of personalised recurrence risk with competing risk of death, multiple visual formats to support patient understanding, and a printed report for shared decision-making**. The iterative co-design process with patients, members of the public, and HCPs enhanced the tool's relevance, usability, and potential acceptability, providing a strong foundation for future testing in real-world clinical settings"

Reviewer: 2
Dr. Nathan Lawrentschuk , University of Melbourne
Comments to the Author:
Overall good paper but please shorten.

We thank the reviewer for their positive assessment of our manuscript.

Also, the bigger question that should be part of this is limiting excessive investigations and follow-up- the minimum follow-up is generally the best. The EAU states for Grade1-2 T1a RCC 3 years, other guidelines say 5- we need clear direction as well as prognosis because this tool could easily mislead patients to wanting more follow-up please comment....

We acknowledge that a key challenge in post-surgical kidney cancer care is balancing adequate surveillance with avoidance of excessive investigations and that clear guidance for different patients is required. The PREDICT-Kidney tool provides a way of

communicating personalised estimates of recurrence risk. The tool aims to improve the communication of the risk of recurrence alongside the competing risk of death from other causes to enable more informed shared-decisions around surveillance and to minimise anxiety amongst those patients recommended to have less intense surveillance.

Moreover, currently, there is no evidence of which follow-up schedule is optimal, and multiple guidelines with slightly different schedules are used. Working on identifying optimal follow-up schedules and potentially reducing consultations is something that is of interest to the research team, but outside the scope of this study.

To make this clearer, we have added the following text to the discussion (line 364):

"A feasibility study is being conducted in three hospital centres across the UK, which will test the acceptability and usability of the tool within its intended clinical pathway. The feasibility study will recruit a larger group of patients and clinicians and aims to include a more diverse group of patients (1). **In the longer-term, we hope to also expand the tool to include information on the individualised benefits and harms of surveillance and adjuvant treatment to support shared-decision making and reduce unnecessary investigations**."

Surely AI has a role here rather than entering of data?

We agree that AI has the potential to automate or assist data entry and risk calculations in online tools in some scenarios. However, the current version of PREDICT-Kidney was designed as a prototype focused on co-design and usability testing with patients and clinicians. Manual entry of patient-specific data allows participants to engage directly with the tool, understand how their inputs affect risk estimates, and provide meaningful feedback on the communication of results.

We acknowledge that future versions of the tool should consider integrating AI-assisted data entry or pre-population from electronic health records to streamline use in clinical practice. This will be a focus of upcoming feasibility and implementation studies.

Also needs to lose 400 words or so to make it punchier.

We thank the Reviewer for the suggestion. We have reduced the manuscript by 500 words.

Bibliography:

1. Laouris P, et al Feasibility study of using the PREDICT kidney tool for patients with localised renal cell carcinoma. BJUI Compass. 2025 Mar 30;6(4):e70014
